# Functional and postoperative outcomes after high-intensity interval training in lung cancer patients: A systematic review and meta-analysis

**DOI:** 10.3389/fonc.2022.1029738

**Published:** 2023-01-20

**Authors:** Zihao Chen, Junqiang Jia, Dongmei Gui, Feng Liu, Jun Li, Jiayuan Tu

**Affiliations:** ^1^ College of Physical Education, Yangzhou University, Yangzhou, China; ^2^ School of Athletic Performance, Shanghai University of Sport, Shanghai, China; ^3^ Department of Orthopedics, Affiliated Hospital of Yangzhou University, Yangzhou, China; ^4^ Department of Gastroenterology, Jining No. 1 People’s Hospital, Jining, China; ^5^ Training Department, Nanjing Sport Institute, Nanjing, China; ^6^ School of Nursing and School of Public Health, Yangzhou University, Yangzhou, China

**Keywords:** HIIT, high-intensity interval training, lung cancer, postoperative outcome, lung function

## Abstract

**Objective:**

The study evaluated the effects of high-intensity interval training (HIIT) on postoperative complications and lung function in patients with lung cancer compared to usual care.

**Methods:**

We searched electronic databases in April 2022, including PubMed, Embase, the Cochrane Library, Web of Science, and the China National Knowledge Infrastructure (CNKI). Two authors independently applied the Cochrane Risk of Bias tool to assess the quality of RCTs. The postoperative complications, length of hospitalization, and cardiopulmonary functions from the studies were pooled for statistical analysis.

**Results:**

A total of 12 randomized controlled trials were eligible for inclusion and were conducted in the meta-analysis. HIIT significantly increased VO_2peak_ (MD = 2.65; 95% CI = 1.70 to 3.60; *I^2^* = 40%; *P <*0.001) and FEV1 (MD = 0.12; 95% CI = 0.04 to 0.20; *I^2^* = 51%; *P* = 0.003) compared with usual care. A subgroup analysis of studies that applied HIIT perioperatively showed significant improvement of HIIT on FEV1 (MD = 0.14; 95% CI = 0.08 to 0.20; *I^2^* = 36%; *P <*0.0001). HIIT significantly reduced the incidence of postoperative atelectasis in lung cancer patients compared with usual care (RD = −0.16; 95% CI = −0.24 to −0.08; *I^2^* = 24%; *P <*0.0001). There was no statistically significant effect of HIIT on postoperative arrhythmias (RD = −0.05; 95% CI = −0.13 to 0.03; *I^2^* = 40%; *P* = 0.22), length of hospitalization (MD = −1.64; 95% CI = −3.29 to 0.01; *P* = 0.05), and the six-minute walk test (MD = 19.77; 95% CI = −15.25 to 54.80; *P* = 0.27) compared to usual care.

**Conclusion:**

HIIT may enhance VO_2peak_ and FEV1 in lung cancer patients and reduce the incidence of postoperative atelectasis. However, HIIT may not reduce the incidence of postoperative arrhythmia, shorten the length of hospitalization, or improve the exercise performance of patients with lung cancer.

**Systematic review registration:**

PROSPERO, CRD42022335441

## 1 Introduction

According to global cancer statistics, lung cancer is one of the most diagnosed cancers, with an estimated 2.2 million new cases and 1.8 million deaths in 2022 (1). Smoking is the major cause of lung cancer, with about 80% to 90% of lung cancer cases related to smoking (2, 3). Lung cancer is divided into small-cell lung cancer (SCLC) and non-small-cell lung cancer (NSCLC), and the prevalence of NSCLC is higher, accounting for about 85% (4). There are many treatments for lung cancer, such as surgical resection, chemotherapy, and radiotherapy (5). Surgical intervention is most applicable to early-stage lung cancer diagnoses and is considered the best curative option (6). Complications adversely affect survival after lung cancer surgery. Fernandez et al. showed that complications including delirium, blood transfusion, reintubation, and pneumonia are associated with worse survival in the early period (0–180 days) (7). Respiratory problems were found to be the most common cause of lung cancer readmission within 30 days after surgery, and postoperative pulmonary complications were strongly associated with mortality within 90 days after surgery (8). A prospective observational study found that patients who underwent a lung resection with postoperative pulmonary complications had a significantly prolonged length of hospital stay post-surgery and reduced overall survival in months compared with patients without postoperative pulmonary complications (9).

Exercise has been found to be effective in improving the health condition, quality of life, and exercise capacity of patients with lung cancer after surgery (10, 11). High-intensity interval training (HIIT) is a unique training method that consists of short (<45 s) to long (2–4 min) physical activity at submaximal to all-out intensity, interspersed with passive or active recovery sessions (12). HIIT was initially used as a physical training method for athletes to improve cardiopulmonary function and has been gradually applied in the field of disease prevention and rehabilitation recently (13). Previous meta-analyses focused on the effect of HIIT on cardiorespiratory fitness in lung cancer patients, especially on peak oxygen uptake (VO_2peak_) (14, 15). There is a lack of studies on postoperative complications, length of hospitalization, and other cardiopulmonary function indicators in lung cancer patients (14). Therefore, in our meta-analysis, we evaluated the effects of HIIT on postoperative complications and lung function in patients with lung cancer compared to usual care.

## 2 Methods

This systematic review and meta-analysis strictly followed the guidelines of the Preferred Reporting Items for Systematic Reviews and Meta-Analyses (PRISMA) guidelines (16). We registered a protocol for this systematic review and meta-analysis on PROSPERO (registration ID is CRD42022335441).

### 2.1 Search strategy and information source

We conducted a literature search on April 23, 2022. Five electronic databases were searched, including PubMed, Embase, Cochrane Library, Web of Science, and China National Knowledge Infrastructure (CNKI).

### 2.2 Inclusion and exclusion criteria

Identified studies were screened for eligibility if they met the following inclusion criteria: (1) patients who had been diagnosed with lung cancer; (2) the exercise protocol was defined as high-intensity interval training; (3) the HIIT group was compared with usual care or standard care; (4) only randomized controlled trials were included; and (5) all languages were available.

The exclusion criteria were as follows: (1) lack of usual care or standard care; (2) studies with missing data or outliers; (3) repeated publications; and (4) meeting abstracts.

### 2.3 Outcomes

Pulmonary function and postoperative complications were the primary outcomes of this study. Secondary outcomes included the length of hospitalization and the six-minute walk test.

### 2.4 Date extraction

Based on the inclusion and exclusion criteria, data were extracted independently by two authors. The data were extracted from each study using a standard form that included the first author, year, country, number of patients, sex percentage, age, TNM cancer stage, intervention (including HIIT protocol and the timing of HIIT), control, primary outcomes, and secondary outcomes. Any disagreements can be resolved through discussion or by having a third researcher reviewing.

### 2.5 Quality assessment

Two authors independently applied the Cochrane Risk of Bias tool to assess the quality of RCTs. It contains the following aspects to assess random sequence generation, allocation concealment, blinding of participants and personnel, blinding of outcome assessment, incomplete outcome data, selective reporting, and other biases. Any disagreement was solved by consensus or by asking another researcher to reassess.

### 2.6 Statistical analysis

Review Manager 5.4 software was used to analyze the extracted data. For continuous outcomes, we used the mean difference (MD) and their 95% confidence intervals for the study. The risk difference (RD) and their 95% confidence intervals were applied to dichotomous outcomes. The imported data were evaluated for statistical heterogeneity. Heterogeneity was tested using the I^2^ statistic and the Cochrane Q statistic, as recommended by the Cochrane Handbook. I^2^ and the p-value for Q statistics were applied to assess the heterogeneity across included trials, and I^2^ >50.0% or P <0.10 was considered significant heterogeneity (17).

## 3 Results

### 3.1 Study selection and characteristic

**Figure 1:**
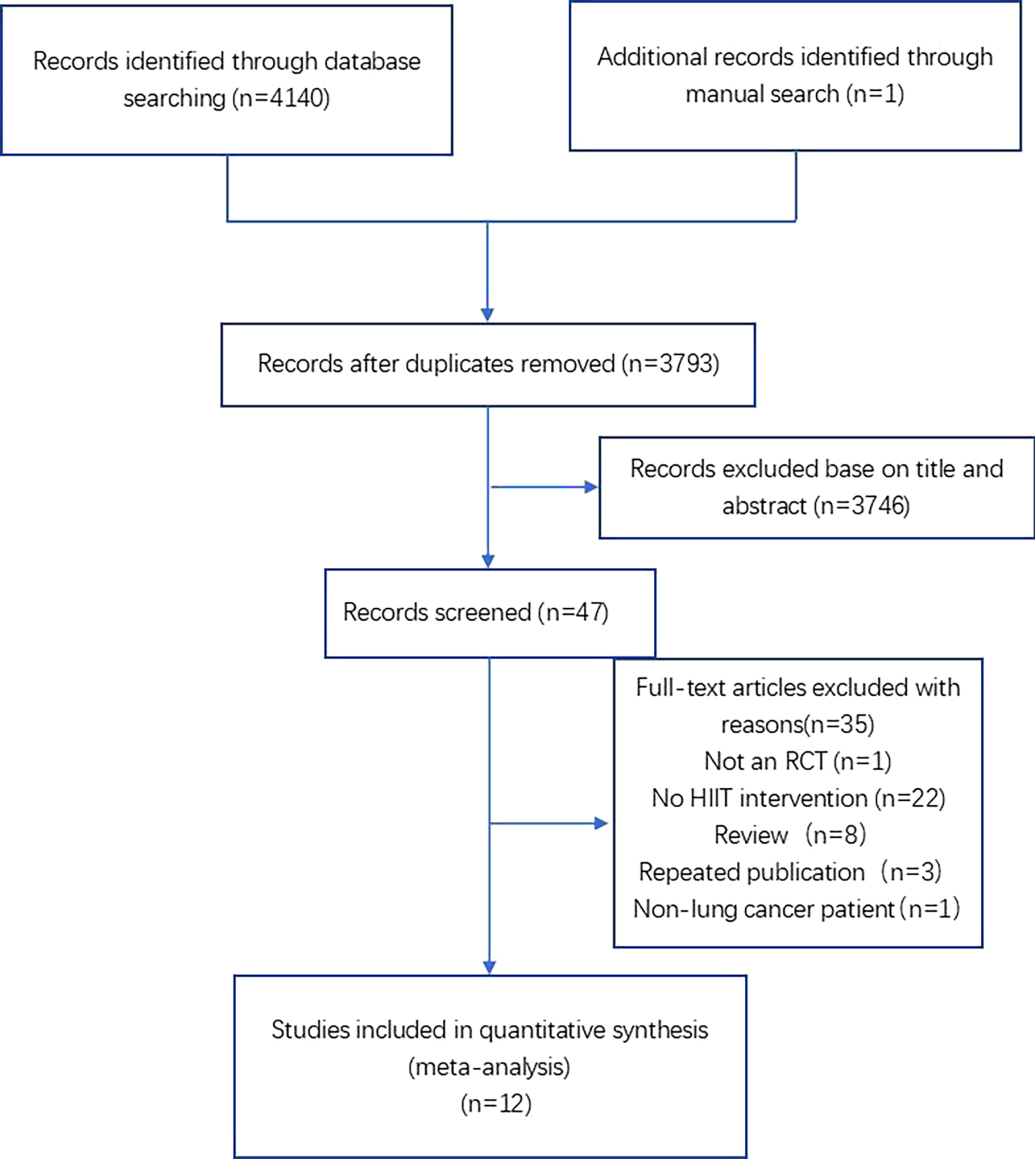
Flow diagram of the selection of studies.

A total of 4,141 articles were retrieved and identified after completing the search strategy, including one additional record identified through a manual search. The number was reduced to 3,793 after removing 348 duplicates. A total of 3,746 articles were excluded by two authors who independently read the title and abstract of each article. After assessing the remaining 47 articles, 32 articles were excluded by screening the full text; the excluded reasons were as follows: no RCT (n = 1), no HIIT intervention (n = 22), review (n = 8), repeated publication (n = 3), and non-lung cancer patient (n = 1). Finally, it resulted in the inclusion of 12 articles (**Figure 1**).

The main characteristics of the HIIT intervention studies included in this review are presented in **Table 1**. The studies originated in China (18–23), Denmark (24, 25), Spain (26), Norway (27), Australia (28), and Switzerland (29). The trial sample size ranged from 15 to 218. Clinical data were collected from 926 lung cancer-related patients in our meta-analysis. The gender of patients was predominantly male (95.5% to 28.6%). However, one study did not accurately report the age and gender characteristics of the patients (25). Two studies recruited patients with non-small-cell lung cancer (NSCLC) or small-cell lung cancer (SCLC) (21, 25). One study enrolled patients with both NSCLS and chronic obstructive pulmonary disease (COPD) (22). The rest of the studies enrolled patients in the early stages of non-small-cell lung cancer (18–20, 23, 24, 26–29).

**Table 1:** Characteristics of included randomized clinical trials.

Study	Country	Patients, total n	Male sex n (%)	Age, years	TNM cancer stage	Intervention	Control	Primary outcomes	Secondary outcomes
Gao (2022)	China	178 (89 vs 89)	56 (63) *vs* 46 (52)	54.36 (10.58) *vs* 52.93 (8.83)	I, II, III NSCLC/SCLC	Post-operative, 3–5 times/day, 3–5 times/week, 2 months Warm-up: no report HIIT: 15–30 s cycling all-out sprint and 15 s rest, total 5–10 min Cool down: no report	Usual care	FEV_1_, FVC, MVV, 6MWT	SAS, FoP-Q-SF, SF-36, TNF-α, CRP, OS
Liu (2017)	China	68 (34 *vs* 34)	17 (50) *vs* 19 (56)	60.8 (4.3) *vs* 62.6 (5.1)	I, II, IIIa NSCLC	Pre-operative, 30 min/times, two times/day, 7 days Warm-up: no report HIIT: 3 ∗(40%–100% HR_max_ climbing stairs and 3 min rest) Cool down: no report	Usual care	FVC, FEV_1_, MVV, DLCO	Postoperative complications
Wang (2020)	China	56 (28 *vs* 28)	12 (42.86) *vs* 13 (46.43)	56.32 (5.48) *vs* 58.04 (8.27)	I, II, III NSCLC	Pre-operative, 1 time/day, 3 months Warm-up: 10 min 70% HR_max_ cycling HIIT: 1 min high intensity (70%–100% HR_max_) cycling + 1 min rest, total 35 min Cool down: 5 min rest	Usual care	6MWT	Dyspnea, anxiety, depression, exercise self-efficacy, self-care ability, Length of hospitalization
Wu (2015)	China	58 (29 *vs* 29)	19 (65.5) *vs* 20 (68.9)	61.4 (6.3) *vs* 61.6 (6.7)	Lung cancer	Pre-operative, five times/week, 2 weeks Warm-up: 1 min 15 W cycling HIIT: 40 min cycling (60%–80% VO_2peak_) and rest (1:1) Cool down: 5 min 15 W cycling	Usual care	Operation time, Intraoperative blood loss, Length of hospitalization	Postoperative complications
Fang (2013)	China	44 (22 *vs* 22)	21 (95.5) *vs* 21 (95.5	64.1 (7.16) *vs* 64.8 (6.82)	I, II, IIIa NSCLC with COPD	Pre-operative, five times/week, 2 weeks Warm-up: 1 min 15 W cycling HIIT: 40 min cycling (60%–80% VO_2peak_) and rest (1:1) Cool down: 5 min 15 W cycling	Usual care	FVC, FEV_1_, MVV, DLCO	WR_peak_, VO_2peak_, AT, HR_max_, VO_2_/HR, VE_max_, Postoperative complications, Length of hospitalization
Licker (2017)	Switzerland	151 (74 *vs* 77)	41 (55) *vs* 50 (65)	64 (13) *vs* 64 (10)	IIIa NSCLC or less	Pre-operative 8 HIIT sessions for 25 Days, three times a week Warm-up: 5 min cycling at 50% W_peak_ HIIT: 2 ∗ (15 s sprints at 100% W_peak_ and 15 s rest), total 20 min Cool down: 5 min cycling at 30% W_peak_	Usual care	Postoperative complications	6MWT, VO_2peak_, Length of hospitalization
Edvardse (2015)	Norway	61 (30 *vs* 31)	13 (43) *vs* 15 (48)	64.3 (9.3) *vs* 65.9 (8.5)	I–IV NSCLC	Post-resection, starting 5–7 weeks after surgery 60 min, three times/week, 20 weeks HIIT: Walking uphill on a treadmill 80%–95% HR_max_ Resistance training: 3*6–12 RM upper and lower limb, back strength	Standard postoperative care	VO_2peak_	FEV_1_, MVV, Tlco, Leg press (1 RM), Hand grip (1 RM), BMI, Total muscle mass, Chair stand, Stair run
Egegaard (2019)	Denmark	15 (8 *vs* 7)	3 (37.5) *vs* 2 (28.6)	64 (5.8) *vs* 65 (5.7)	IIIa, IIIb, IV NSCLC	During radiotherapy, 20 min/times, five times/week, 7 weeks Warm-up: 5 min light cycling HIIT: 5 ∗ 30 s 80%–95% iPPO cycling and 30 s rest, total 10 min Aerobic training: 5 min 80% iPPO cycling	Standard care	Feasibility	VO_2peak_, 6MWT, FEV_1_, FACT-L, HADS, reported daily, blood pressure
Cavalheri (2017)	Australia	17 (9 *vs* 8)	5 (29)	66 (10) *vs* 68 (9)	I, II, IIIa NSCLC	Post-resection 6–10 weeks or post-chemotherapy 4–8 weeks, 60 min/times, three times/week, 8 weeks Warm-up: no report HIIT: 20 min walking 70/80% 6MWT speed or 10 min cycling 80% WR_peak_ Resistance training: 3 ∗ 10 upper limb training Cool down: no report	Usual care	VO_2peak_, 6MWT, AT	SF-36, FACT-L, EORTC QLQ-C30, muscle strength, respiratory function (spirometry)
Hwang (2012)	China	24 (13 *vs* 11)	5 (38.5) *vs* 7 (63.6)	61 (6.3) *vs* 58.5 (8.2)	IIIa, IIIb, IV NSCLC	During targeted therapy, 30–40 min, three times/week, 8 weeks Warm-up: 10 min running or cycling HIIT: treadmill or cycling ergometer 80% VO_2peak_/RPE 15–17 and 60% VO_2peak_ active recovery, total 25 min Cool down: 5 min	Usual care	VO_2peak_, RER	EORTC QLQ-C30
Messaggi-Sartor (2019)	Spain	37 (16 *vs* 21)	8 (50) *vs* 18 (85.7)	64.2 (8.1) *vs* 64.8 (8.9)	I or II NSCLC	Post-operative, 1 h/time, three times/week, 8 weeks Warm-up: 5 min HIIT: IEMT (5 ∗ 10 breathing) 50%PImax/PEmax (15 min) Aerobic training: cycling 60% W_peak_ (30 min) Resistance training: bicep curl, chest, and shoulder press Cool down: 5 min	Post-operative standard care	VO_2peak_	Respiratory muscle strength, IGF-I, IGFBP-3, EORTC QLQ-C30, lung cancer recurrence, death Length of hospitalization
Quist (2020)	Denmark	218	No report	>18	IIIb–IV NSCLC and SCLC ED	During chemotherapy, 90 min/times, two times/week, 12 weeks Warm-up: 10 min 60%–90% HR_max_ cycling Strength training: 3 ∗ 5–8 70%–90% 1RM upper and lower training HIIT: interval training on stationary bikes, 85%–95% HR_max_, 10–15 min Cool down: 20–30 min stretching and progressive relaxation	Usual care	VO_2peak_	Muscle strength (1RM), 6MWT, FEV_1_, HADS, FACT-L

NSCLC, non-small cell lung cancer; SCLC, small cell lung cancer; FEV1, forced expiratory volume in 1 s; FVC, forced vital capacity; MVV, maximal voluntary ventilation; 6MWT, six-minute walk test; SAS, Self-rating Anxiety Scale; FoP-Q-SF, Fear of Progression Questionnaire-Short Form; SF-36, the 36-Item Short Form Health Survey; TNF-α, Tumor necrosis factor-α; CRP, C-reactive protein; OS, Overall survival; DLCO, carbon monoxide diffusing capacity; WR_peak,_ peak work rate; VO_2peak,_ peak oxygen uptake; AT, anaerobic threshold; HR_max_, maximal heart rate; VO_2_/HR, oxygen pulse; VE_max_, maximal minute ventilation; Tlco, carbon monoxide transfer factor; RM, repetition maximum; FACT-L, Functional Assessment of Cancer Therapy-Lung; HADS, the Hospital Anxiety and Depression Scale; EORTC QLQ-C30, The European Organization for Research and Treatment of Cancer, Quality of Life Questionnaire Core 30; RER, respiratory exchange rate; IGF-I, levels of serum insulin growth factor I; IGFBP-3, IGF binding protein 3; HIIT, high-intensity interval training; IEMT, inspiratory and expiratory muscle training; iPPO, patient’s peak Max power; HR_max_, maximal heart rate; IEMT consisted of sets of repetitions followed by 1–2 min of unloaded recovery breathing (off the device), twice a day, 3 days per week, for 8 weeks; PImax, maximal inspiratory pressure; PEmax, maximal expiratory pressure; W_peak_, peak workload.

Five studies conducted HIIT interventions preoperatively (18–20, 22, 29). In contrast, three studies conducted HIIT interventions postoperatively (21, 26, 27). One study applied HIIT during post-operation or post-chemotherapy (28). Three studies applied HIIT programs during targeted therapy (23), radiotherapy (24), and chemotherapy (25), respectively.

Most studies used high-intensity interval bicycling as the intervention method; a few studies used high-intensity interval walking; and a study used high-intensity interval respiratory muscle training (26). Seven studies applied high-intensity interval training solely (18–23, 29). Three studies combined HIIT with resistance training (25, 27, 28). One study combined HIIT with aerobic training (24). In particular, one study combined high-intensity interval respiratory muscle training, resistance training, and aerobic training together (26).

### 3.2 Risk of bias

**Figure 2:**
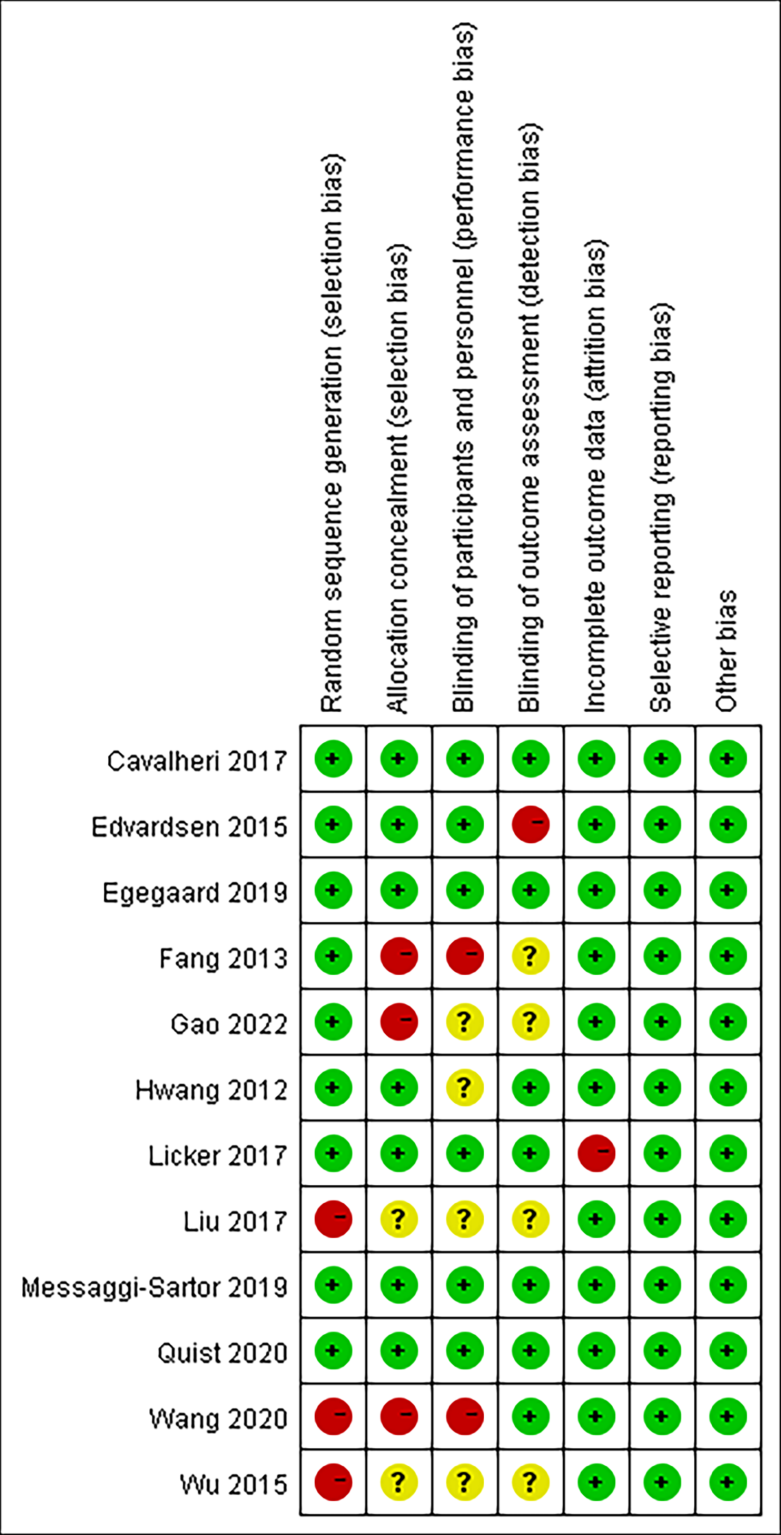
Risk of bias summary review authors’ judgments about each risk of bias item for each included study.

(**Figure 2**) presents the risk of bias assessments for all 12 included studies. Four studies were deemed to have a low risk of bias (24–26, 28), and eight studies were considered to have a moderate risk of bias (18–23, 27, 29).

### 3.3 Outcomes

#### 3.3.1 Pulmonary function

**Figure 3:**
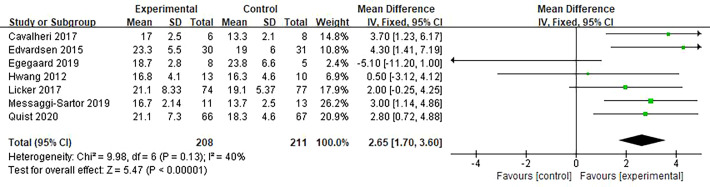
Meta-analysis of the effect of HIIT on VO_2peak_ (ml/kg/min) among lung cancer patients.

**Figure 4:**
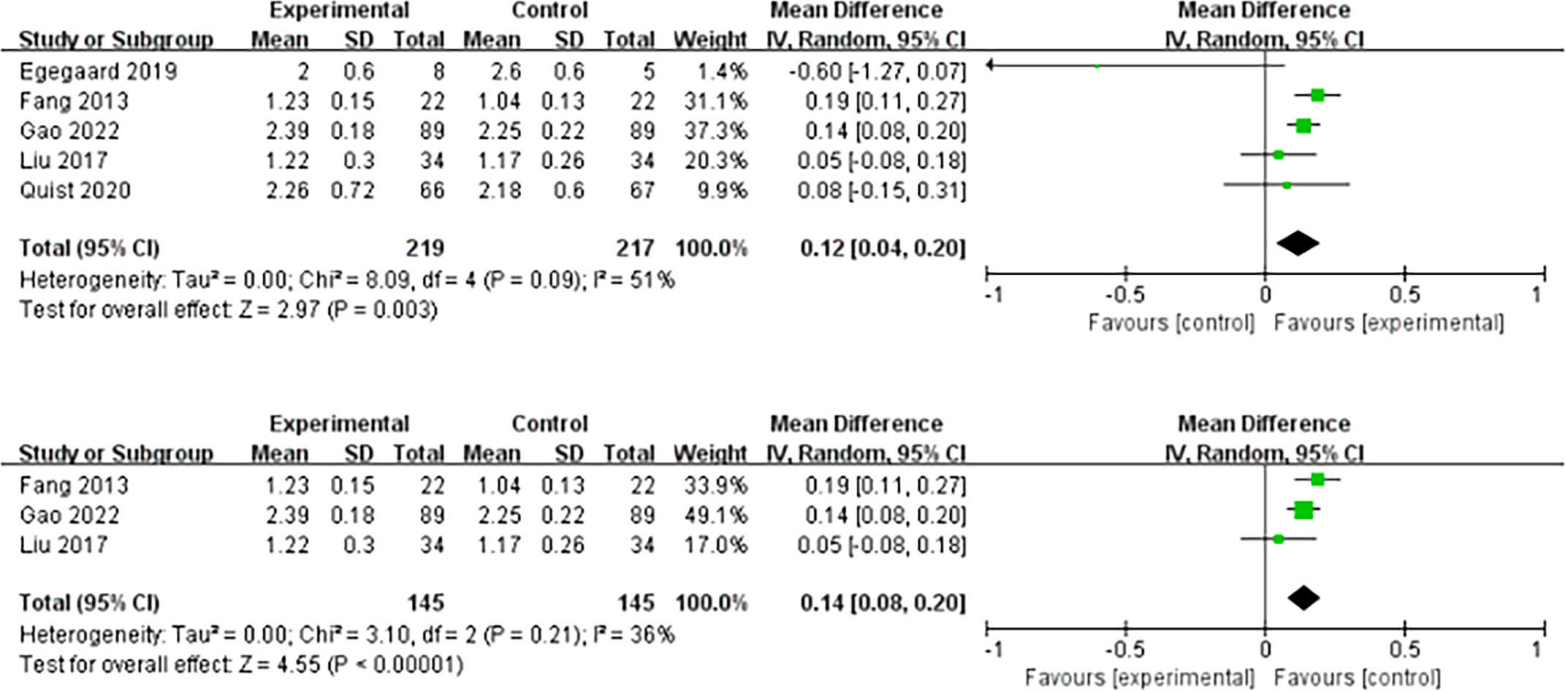
Meta-analysis of the effect of HIIT on FEV1 (L) and subgroup analysis among lung cancer patients.

Seven studies supported the finding that HIIT increased VO_2peak_ in patients with lung cancer. The result of the meta-analysis was statistically significant and favored the HIIT intervention (MD = 2.65; 95% CI = 1.70 to 3.60; *I^2^* = 40%; *P <*0.001) (**Figure 3**). Five studies examined the effect of HIIT intervention on forced expiratory volume in 1 s (FEV1). The results showed statistical significance and favored the HIIT intervention (MD = 0.12; 95% CI = 0.04 to 0.20; *I^2^* = 51%; *P* = 0.003). We conducted a subgroup analysis to solve the heterogeneity. Only studies that conducted HIIT perioperatively were included (20–22). The results still support that HIIT benefits FEV1 among patients with lung cancer compared with usual care (MD = 0.14; 95% CI = 0.08 to 0.20; *I^2^* = 36%; *P <*0.0001) (**Figure 4**).

#### 3.3.2 Postoperative complications

**Figure 5:**
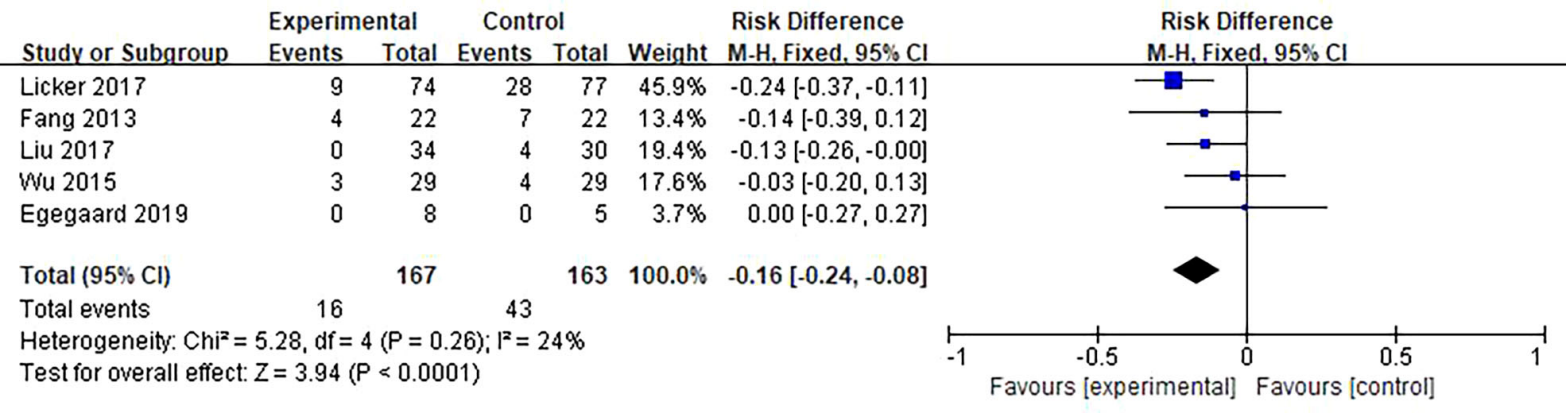
Meta-analysis of the effect of HIIT on atelectasis among lung cancer patients.

**Figure 6:**
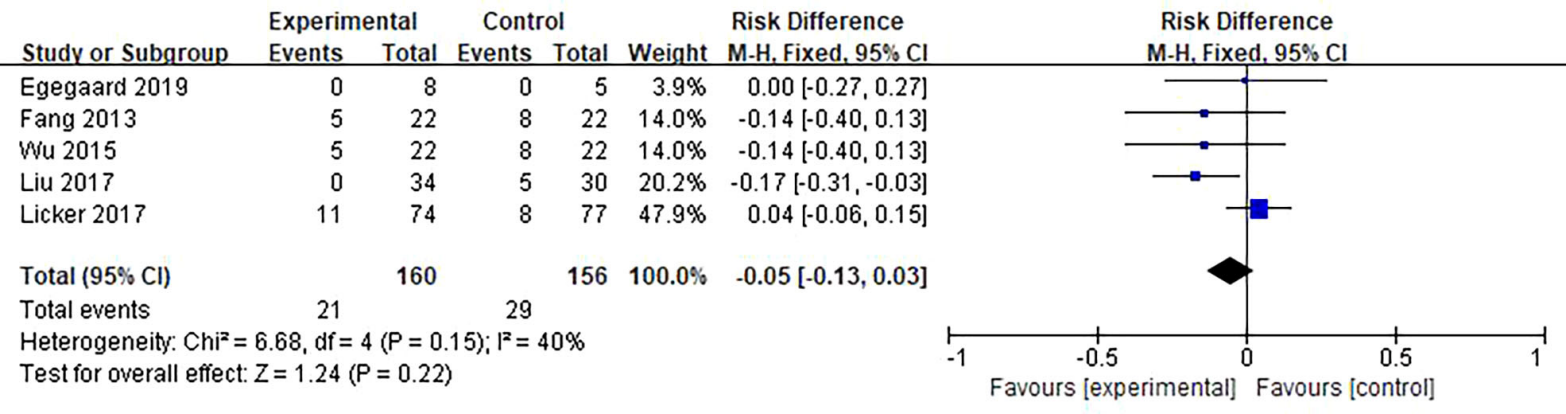
Meta-analysis of the effect of HIIT on arrhythmias among lung cancer patients.

A total of 59 patients in five studies reported atelectasis events, and the results showed that the HIIT intervention was beneficial to reduce postoperative atelectasis events in lung cancer patients compared with usual care (RD = −0.16; 95% CI = −0.24 to −0.08; *I^2^* = 24%; *P <*0.0001) (**Figure 5**). Five studies reported arrhythmia events among 50 patients. There was no effect that HIIT had on postoperative arrhythmias in lung cancer patients compared to usual care (RD = −0.05; 95% CI = −0.13 to 0.03; *I^2^* = 40%; *P* = 0.22) (**Figure 6**).

#### 3.3.3 Length of hospitalization

**Figure 7:**
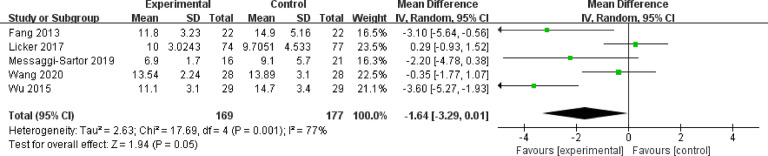
Meta-analysis of the effect of HIIT on length of hospitalization (days) among lung cancer patients.

Five studies with 346 patients who reported length of hospitalization were included in our meta-analysis. The results showed no significant difference between the HIIT group and the usual care group (MD = −1.64; 95% CI = −3.29 to 0.01; *P* = 0.05). Heterogeneity was high (*I^2^* = 77%) (**Figure 7**).

#### 3.3.4 The six-minute walk test

**Figure 8:**
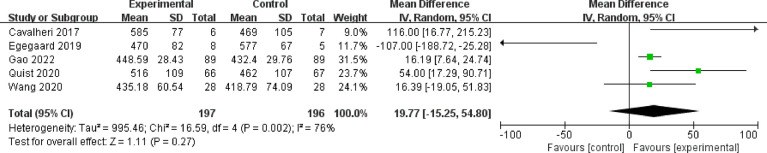
Meta-analysis of the effect of HIIT on 6MWT (meters) among lung cancer patients.

In our study, five studies presented 393 patients’ 6MWT performance. The results proved no difference between the HIIT group and the usual group (MD = 19.77; 95% CI = −15.25 to 54.80; *P* = 0.27). Heterogeneity was high (*I^2^* = 76%) (**Figure 8**).

## 4 Discussion

This study examines the functional and postoperative outcomes of a high-intensity interval training intervention in lung cancer patients. With regard to pulmonary function, our results showed that both VO_2peak_ and FEV1 improved with the application of HIIT among lung cancer patients. With regard to postoperative outcomes, the postoperative incidence of atelectasis was significantly reduced. However, there is limited evidence that HIIT does not reduce the incidence of arrhythmias. Due to the high heterogeneity of the results, it is still unclear whether the length of hospitalization was shortened, and the six-minute walk performance increased in lung cancer patients.

Previous studies mentioned that VO_2peak_ played a key role in predicting surgical outcomes and survival in NSCLC patients (30, 31). It has been reported that HIIT induces VO_2peak_ enhancement (32). A meta-analysis that included 305 lung cancer patients from eight studies showed that VO_2peak_ was significantly increased by HIIT compared to usual care (14). Another study demonstrated that HIIT had a greater impact on VO_2peak_ than usual care (15). The same results were also observed in our meta-analysis. However, the difference is that our study focused not only on lung function in lung cancer patients after HIIT rehabilitation but also on postoperative outcomes. Our results showed that HIIT could also effectively improve FEV1 and reduce the postoperative incidence of atelectasis. Interestingly, our study showed that HIIT did not reduce the incidence of arrhythmia, possibly due to postoperative arrhythmia being associated with surgical inflammation, autonomic nerve injury, and cardiac overload (33). More research is still needed.

HIIT can effectively increase muscle metabolic capacity and promote increases in muscle strength and hypertrophy, thus improving lung respiratory function and mobility in lung cancer patients (27, 28). A study demonstrated that HIIT may increase skeletal muscle mitochondrial capacity, leading to improvements in whole-body metabolic homeostasis by improving several classical markers of mitochondrial biogenesis, including the maximal activity of citrate synthase (CS) and cytochrome c oxidase (COX) as well as the total protein content of CS and COX subunits II and IV (34). Another study found that six sessions of HIIT expanded skeletal muscle mitochondria, as assessed by cytochrome c oxidase activity (35). In terms of exercise capacity, cancer-induced cachexia causes muscle atrophy in cancer patients by inhibiting muscle protein synthesis and enhancing muscle catabolism (36). In two rat models, researchers found that HIIT could lead to muscle hypertrophy by improving the IGF-I/Akt/FoxO and myostatin/Smad signal transduction pathways (37), activating the mTOR pathway, altering the expression of MuRF-1 and MAFbx proteins, and improving autophagic flux (38).

The benefits of HIIT may be influenced by the timing of exercise. Perioperative exercise training included preoperative exercise, acute post-operative (in-hospital) exercise, and postoperative exercise (39). It has been indicated that lung cancer patients who have undergone resection can benefit from preoperative exercise, which includes the improvement of both pulmonary function and exercise capacity, a lower incidence rate of postoperative complications, a shorter length of hospital stay, and a lower degree of dyspnea (40–42). Acute post-operative exercise (in-hospital) involves sitting out of bed and walking around the hospital ward, with the aim of discharge from the hospital as soon as possible (43, 44). There is no consensus on whether acute post-operative exercise improved post-operative physical activity level, mobility, or lung function (45, 46). Cavalheri et al. found that postoperative exercise enhanced exercise performance but not HRQoL and FEV1 in patients with lung cancer (47). Interestingly, a randomized controlled trial showed that early rehabilitation avoided a temporary decline in HRQoL by comparing the effect of early rehabilitation (14 days after surgery) with the late rehabilitation group (14 weeks after surgery) (48). It can be inferred that preoperative HIIT is more beneficial for patients with lung cancer (10). However, there are few studies on preoperative HIIT, and further prospective studies with large samples are needed to explore the benefits of preoperative HIIT. Limited evidence has suggested that exercise training enhances mobility and physical fitness in lung cancer patients during chemotherapy (49). Larger randomized controlled trials are warranted to prove the effect of combining exercise with targeted therapy, chemotherapy, or radiotherapy.

The exercise type of HIIT may also influence the rehabilitation of lung cancer patients after surgery. Most studies included in this meta-analysis used cycling as a type of high-intensity training. Only one study used respiratory muscle training as a type of high-intensity training (26). Laurent et al. (50) showed that lung cancer patients who accepted resection surgery decreased pulmonary postoperative complications by applying respiratory muscle training. However, further studies are needed to determine which exercise type HIIT is more favorable for patients with lung cancer. Meanwhile, the stage, subtype, and smoking habits of patients with lung cancer may also affect postoperative rehabilitation and should be considered. Considering safety and practicality, it is possibly harmful to apply HIIT at all-out intensity for individuals with severe disease (34). Low-volume HIIT is safer and has a similar improvement effect as high-volume HIIT in improving individual cardiopulmonary function (51). Low-volume HIIT is defined as repetitions range from 1 to 10 times with an active interval time of fewer than 15 min, whereas high-volume HIIT requires active intervals and repetitions of more than 15 min and four times, respectively (52). Seven studies included in our meta-analysis used low-volume HIIT (20, 21, 23–26, 29). Therefore, we assume that low-volume HIIT may be a safer way to treat patients with lung cancer, and this needs to be confirmed. In this meta-analysis, three included studies combined HIIT with resistance training (25, 27, 28), and one combined HIIT with aerobic training (24). It is still unclear whether HIIT combined with other training will have a larger effect on lung cancer patients than using HIIT solely.

## 5 Conclusion

In conclusion, HIIT improved pulmonary function and reduced postoperative atelectasis in patients with lung cancer. However, the incidence of postoperative arrhythmias was not decreased by HIIT. Due to high heterogeneity, shortening the length of hospitalization and enhanced exercise capacity in lung cancer patients after HIIT intervention were not supported in this meta-analysis.

## 6 Future directions

As a timesaving, effective, and applicable rehabilitation method, high-intensity interval training could open a new perspective for treating lung cancer patients (14). However, the high-intensity interval training protocol remains unclear. It is necessary to determine the optimal types of exercise, the timing of using HIIT, intensity, and interval time in the future. Although some studies have obtained some results, future studies with large samples are still needed. At the same time, the different stages, subtypes, types of surgery, and smoking habits of lung cancer patients should be considered in HIIT rehabilitation, which significantly affects postoperative outcomes. Finally, the mechanism of HIIT for improving cardiopulmonary function and its effect on postoperative outcomes in patients with lung cancer should be more concentrated on by researchers.

## 7 Theoretical and practical implication

HIIT has great potential for clinical rehabilitation of patients with lung cancer. Because of its ease of operation and low economic cost, it can effectively improve the postoperative rehabilitation of lung cancer patients and reduce their economic burden. In clinical practice, clinicians and nurses can integrate HIIT into the treatment process as a beneficial measure to improve the health status of lung cancer patients. Personalized HIIT protocols should be developed based on the different treatment methods and health status of lung cancer patients.

## 8 Limitations

There are some limitations to our study, and further research is needed. First, most of the literature included in our study is a small sample, and the conclusions of these studies need to be treated with caution. Second, lung cancer patients are mainly male, and there may be differences between male and female patients. But we did not study them separately in our study. Third, although the included kinds of literature all adopted high-intensity interval training plans, the intensity and interval time of high-intensity training were different, which might affect the final results. Fourth, cycling was the main type of exercise in most of the included studies, while walking was also used. Bias can also be caused by different types of movement. Finally, warm-up and rest are equally important in HIIT planning, and some studies did not report warm-up and rest programs.

## Data availability statement

The original contributions presented in the study are included in the article/supplementary material. Further inquiries can be directed to the corresponding author.

## Author contributions

ZC and JT conceived and planned the review, assessed the methodologic quality of the studies, verified the data, and drafted and revised the manuscript. JJ assessed the methodologic quality of the studies. DG extracted data. FL and JL conducted the literature search and selected studies. JT provided methodologic advice, content expertise, and revised the manuscript. All authors contributed to the article and approved the submitted version.
